# UTILIZING COMMUNITY CONNECTIONS: A SOCIAL DETERMINANTS OF HEALTH SCREENING FOR COMMUNITY-DWELLING OLDER ADULTS

**DOI:** 10.1093/geroni/igae098.2883

**Published:** 2024-12-31

**Authors:** Kelly O’Sullivan, Raven Weaver, Cory Bolkan, Chad Gotch, Erik Geissal, Breanne Swanson

**Affiliations:** Washington State University, Pullman, Washington, United States; Washington State University, Pullman, Washington, United States; Washington State University, Pullman, Washington, United States; Washington State University, Pullman, Washington, United States; Vancouver Clinic, Vancouver, Washington, United States; Area Agency on Aging & Disabilities of Southwest Washington, Vancouver, Washington, United States

## Abstract

Community-based aging resources, like Area Agencies on Aging (AAA), are often underutilized. Establishing partnerships with healthcare providers can promote better awareness and use of available services/supports. We evaluated a community partnership between a primary care clinic and an AAA; both community partners were committed to reducing adverse health outcomes. This exploratory intervention study assessed social determinants of health (SDH) needs of Medicare-eligible primary care patients (n = 385) during an annual visit (Mage=72.5; 89.6% White) and connected patients to appropriate supportive aging services. There were 284 participants with identified unmet SDH needs, but only 36 participants accepted referral to AAA resources. We analyzed Medicare’s Risk Adjustment Factor (RAF) score at baseline and participants’ use of advanced care (i.e., ER visits/hospital readmissions/institutionalizations), urgent care, and primary care, over a 6-month period. Descriptive, bivariate, and multivariate analyses revealed positive associations between RAF score with age (p< 0.001) and number of SDH needs (p< 0.001), advanced care with SDH needs (p=0.004) and RAF score (p< 0.001), and primary care visits with SDH needs (p=0.003) and RAF score (p< 0.001). The screening revealed that older adults’ SDH needs are often unmet and underrecognized. Cross-sector partnerships between medical and social models of health can reduce risk and meet needs of community-dwelling older adults. Policy provisions that encourage such partnerships are needed to improve community-dwelling older adults’ wellbeing, independence, and ability to age-in-place.

